# Spatially ordered recruitment of fast muscles in accordance with movement strengths in larval zebrafish

**DOI:** 10.1186/s40851-024-00247-8

**Published:** 2025-01-03

**Authors:** Sayaka Shimizu, Taisei Katayama, Nozomi Nishiumi, Masashi Tanimoto, Yukiko Kimura, Shin-ichi Higashijima

**Affiliations:** 1 National Institutes of Natural Sciences, Exploratory Research Center On Life and Living Systems (ExCELLS), National Institute for Basic Biology, Okazaki, Aichi 444-8787 Japan; 2 Graduate University for Advanced Studies (SOKENDAI), Okazaki, Aichi 444-8787 Japan; 3 Division of Biological Science, Graduate School of Science, Nagoya University, Nagoya, 464-8602 Japan

**Keywords:** Zebrafish, Muscle, Movement, Recruitment, Swimming

## Abstract

In vertebrates, skeletal muscle comprises fast and slow fibers. Slow and fast muscle cells in fish are spatially segregated; slow muscle cells are located only in a superficial region, and comprise a small fraction of the total muscle cell mass. Slow muscles support low-speed, low-force movements, while fast muscles are responsible for high-speed, high-force movements. However, speed and strength of movement are not binary states, but rather fall on a continuum. This raises the question of whether any recruitment patterns exist within fast muscles, which constitute the majority of muscle cell mass. In the present study, we investigated activation patterns of trunk fast muscles during movements of varying speeds and strengths using larval zebrafish. We employed two complementary methods: calcium imaging and electrophysiology. The results obtained from both methods supported the conclusion that there are spatially-ordered recruitment patterns in fast muscle cells. During weaker/slower movements, only the lateral portion of fast muscle cells is recruited. As the speed or strength of the movements increases, more fast muscle cells are recruited in a spatially-ordered manner, progressively from lateral to medial. We also conducted anatomical studies to examine muscle fiber size. The results of those experiments indicated that muscle fiber size increases systematically from lateral to medial. Therefore, the spatially ordered recruitment of fast muscle fibers, progressing from lateral to medial, correlates with an increase in fiber size. These findings provide significant insights into the organization and function of fast muscles in larval zebrafish, illustrating how spatial recruitment and fiber size interact to optimize movement performance.

**Supplementary Information:**

The online version contains supplementary material available at 10.1186/s40851-024-00247-8.

## Background

Skeletal muscle in vertebrates consists of two types of muscle fibers: slow and fast [1, 2]. In mammals, the fast muscle population can be further divided into two major subtypes: type FF (fast fatigable) and type FR (fast resistant) [3]. Different types of muscles are innervated by different types of motoneurons, forming motor units. Differential activation of motor units generates movements with varying forces and speeds [4]. Fast motor units are responsible for high-speed movements and/or strong forces, while slow motor units facilitate low-speed movements and/or weaker forces [5, 6].

Faster and stronger motor units tend to be larger in terms of motoneuron size, muscle fiber size, and number of innervating fibers [7, 8]. Research has revealed a size principle of motor control, which relates the order of recruitment of motoneurons and muscle fibers [8, 9]. According to this principle, the pool of active cells increases in size with progressive increases in the force and speed of movement, with stronger or faster motor units being added to the set of motor units active during weaker or slower movements.

**Fig. 1:**
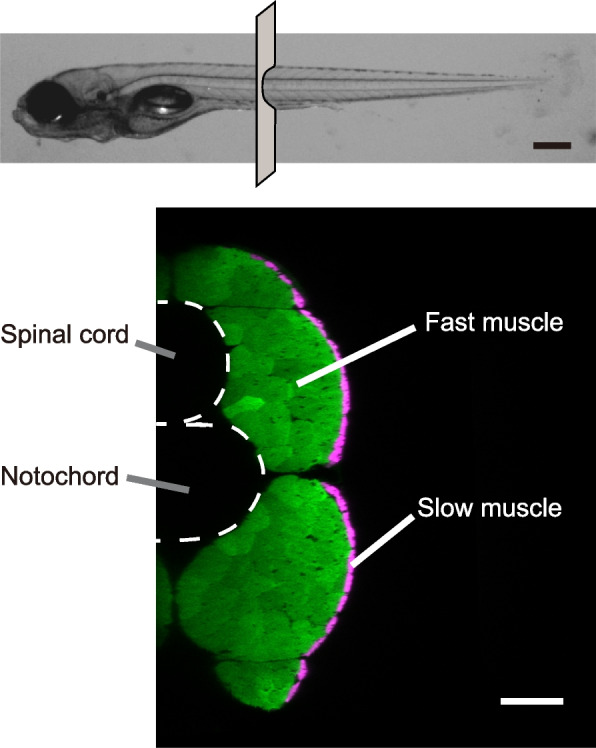
Structure of fast and slow muscles in larval zebrafish. Top panel, lateral view of a 5-dpf larval zebrafish. The position for the cross section in the bottom panel is shown. Scale bar, 300 µm. Bottom panel, cross section view computed from confocal images of a Tg(*actc1b*:GFP); Tg(*smyhc1*:tdTomato) fish. In this compound transgenic fish, GFP (green signal) and tdTomato (magenta signal) are expressed in fast and slow muscles, respectively. Only the right half of muscles is presented. Scale bar, 30 µm

In mammals, fast and slow muscle fibers (and thus muscle cells) are intermingled within a single muscle [7]. In fish trunk muscles, in contrast, slow and fast muscle cells show complete spatial segregation. Slow muscle cells are located only in a superficial region and comprise a small fraction of the total muscle cell mass. The vast majority of trunk muscle mass in fish consists of fast muscle cells (Fig. 1).

The size principle also applies to muscle recruitment during movements in fish. During slow or weak movements, such as slow swimming, only the slow muscles are active. As the speed or strength of the movements increases, fast muscles are recruited [10, 11]. However, the spectrum of movement speed and strength cannot be divided into just two discrete categories; rather, it is continuous. This raises the question of whether recruitment patterns exist within the larger population of fast muscle cells.

The segregated configuration of slow and fast muscles in fish suggests that contracting the superficial region of the body may be mechanically and/or energetically advantageous for producing slow or weak movements. This raises the possibility that laterally located fast muscles are the first to be recruited when slightly more powerful movements are required. Extending this idea further, there may be spatially ordered recruitment patterns within fast muscles, with recruitment progressing from lateral to medial regions as movement speed or strength increases. Indeed, one study in adult rainbow trout supports this idea, demonstrating that superficially located fast muscles are active during fast swimming, while those in the most medial region are inactive during this activity, becoming active only during escape responses, the strongest movements a fish can produce [12].

Neuroanatomical studies, along with physiological investigations in the motoneurons of larval zebrafish, also support the notion of spatially ordered recruitment patterns in muscles [13–15]. These studies show a spatial order in the innervation territories among motoneurons based on their sizes. The largest motoneurons, involved in fast and strong movements, innervate the entire territory of fast muscles from medial to lateral. As motoneuron size decreases (accompanied by a reduction in speed or strength of the movements they control), their innervation territory tends to be more confined to the lateral region of the muscle mass. While these studies suggest the existence of spatially ordered recruitment patterns in fast muscles, no direct, systematic analyses of recruitment patterns during various speeds and strengths of movements have been reported to date.

In the present study, we examined action intensity-specific fast muscle recruitment patterns in larval zebrafish by calcium imaging and electrophysiology. Results from both methods support the idea of the spatially ordered recruitment patterns in fast muscles.

## Results

### Calcium imaging of fast muscles during movements of various strengths

**Fig. 2:**
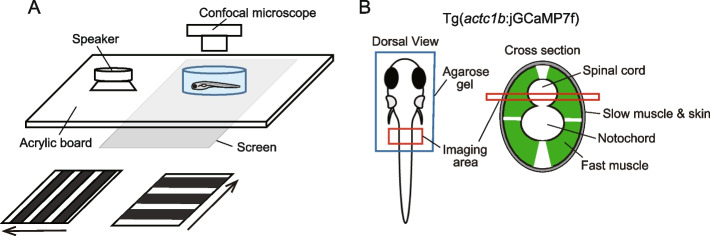
Schematic illustration of the method for calcium imaging in fast muscles. **A** Schematic illustration of the imaging setup. A larval fish, mounted in agarose, is positioned in a petri-dish. The dish is placed on a plate with an attached audio speaker to deliver vibration stimuli, which elicit escape movements. A screen located beneath the plate provides visual stimuli. Specifically, forward-moving gratings elicit slow swimming (shown at bottom left), while laterally moving gratings induce turn movements (shown at bottom right). Fast muscles in the mid trunk region are observed dorsally using a confocal microscope. **B** Schematic illustrations for imaging fast muscles. The fish express jGCaMP7f, a genetically-encoded calcium indicator, in fast muscles. The left panel provides a dorsal view, while the right panel presents a cross-sectional view. In the dorsal view, the rostral part of the body is immobilized in agarose, allowing free movement of the tail. The red-colored rectangle represents the imaging field. In the cross-sectional view, green coloring represents fast muscles. The red rectangle indicate the depth of the optical section captured during confocal imaging

To examine spatial activation patterns of fast muscles during behaviors of different strengths, we generated Tg(*actc1b*:jGCaMP7f) transgenic fish, which expressed the genetically-encoded calcium indicator jGCaMP7f [16] in fast muscles. (Note that the *actc1b* promoter drives reporter gene expression only in fast muscles, not in slow muscles; Fig. 1). The larval fish of Tg(*actc1b*:jGCaMP7s) at 5 dpf (days after post fertilization) was mounted on a petri dish with agarose gel. The dish was placed on a plate with an attached audio speaker to deliver vibration stimuli (Fig. 2A). Additionally, a screen was positioned beneath the plate to present visual stimuli (moving gratings) via a projector (Fig. 2A and Additional file 1). The plate was placed under a confocal microscope to observe the muscles vertically (Fig. 2B). The tail of the fish was freed from the agarose, allowing us to capture tail movements from below using a high-speed camera (Fig. 2B and Additional file 1).

We aimed to analyze spatial activation patterns of fast muscles during three different types of movements: (1) escapes evoked by sudden vibration stimuli [17–19], (2) turns evoked by visual stimuli (laterally-moving gratings) [20], and (3) slow swimming prompted by visual stimuli (forward-moving gratings) [20]. Previous behavioral studies have demonstrated that the angular-velocities of the tail movements differ significantly among these movement types [21].

We first confirmed whether our setup could reliably evoke the expected movements. The sudden vibration stimulus consistently elicited strong tail movements (Additional file 2A), indicating successful evocation of escape responses. Visual stimuli with laterally moving gratings occasionally produced large tail flips (Additional file 2B), suggesting the occurrence of turning movements. The angular velocities of the tail flips were smaller than those of escape movements (Additional file 2D). Visual stimuli with forward-moving gratings often triggered bouts of slow swimming, characterized by periodic tail oscillations lasting several hundred milliseconds. The angular velocities for tail movements evoked by forward-moving gratings were the smallest among the three movement types (Additional file 2C and 2D). These results demonstrate that our apparatus successfully evoked escape, turn, and slow swimming movements. Based on the observed angular velocities, we conclude that the strength of trunk muscle activation follows this order: escapes, turns, and slow swimming.

**Fig. 3:**
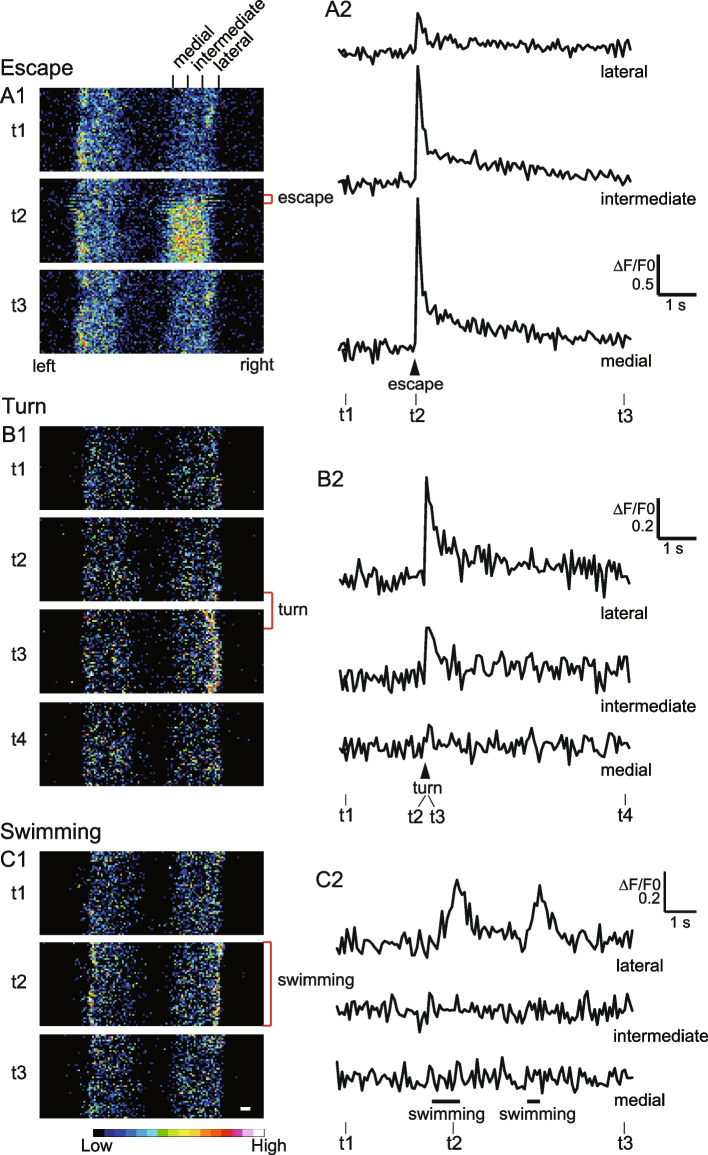
Fluorescence responses in fast muscles during escape, turn, and swimming behaviors. **A1** Pseudocolored images show fluorescence in fast muscles during an escape. Top panel (t1): Baseline image before the escape. Middle panel (t2): Image during escape movement. The fish displays an escape body bend to the right during the scan period indicated by the red bracket. Movement-induced displacement is apparent in some scan lines. Bottom panel (t3): Image when the fluorescence returned to near baseline post-escape. Muscles are divided into three sections along the medio-lateral axis for regional fluorescence quantification (indicated at the top). **A2** Plots of fluorescence responses in each region of the right muscle from **A1**. Timepoints corresponding to images in **A1** are marked as t1, t2, and t3. **B1** Similar to **A1** but showing a turn movement to the right. The turn begins at the end of the second frame (t2) and spans two frames (t2 and t3) with a duration of ~ 30 ms (indicated by the red bracket). **B2** Plots of fluorescence responses in each region of the right muscle from **B1**. Timepoints corresponding to **B1** images are marked as t1, t2, t3, and t4. **C1** Similar to **A1** but for swimming. The fish swims throughout the period of the second frame (t2) (indicated by the red bracket). **C2** Plots of fluorescence responses in each region of the right muscle from **C1**. Timepoints for images in **C1** are marked as t1, t2, and t3. Scale bar, 10 µm

Figure 3 A1 shows a representative example of the calcium imaging experiments for the escape movements. Both the left and right muscles along with the spinal cord in segments 12–14 were imaged (see Fig. 2B). The top panel (t1) shows an image before an escape. The middle panel (t2) shows an image at which an escape movement occurred. Note that the X–Y image from the confocal microscope consists of horizontal scan lines (X-axis) that sequentially moves from the top (rostral) to the bottom (caudal). The escape movement was initiated at the time when the top ~ 20% of the frame had been scanned (indicated as “escape”). Upon the escape movement, large fluorescent intensity change was observed in the wide range of the right muscles, indicating activation of the entire fast muscle region. The bottom panel (t3) shows an image when fluorescence intensity of the right muscle returned to near baseline level.

**Fig. 4:**
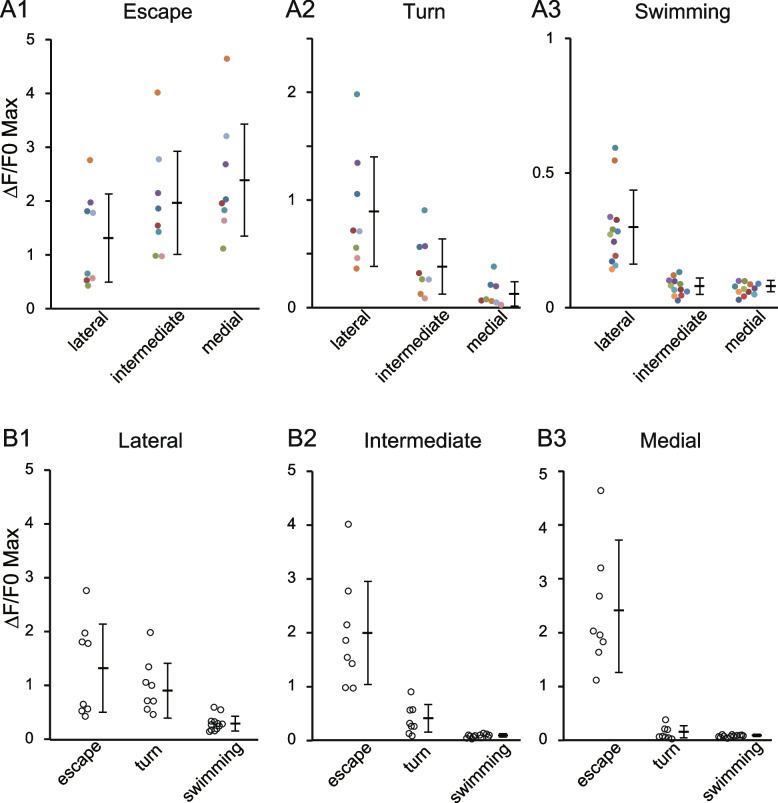
Fluorescence responses in each section of fast muscles during escape, turn, and swimming movements. **A1-A3** Each panel represents data for a specific movement type: Escape **A1**, Turn **A2**, and Swimming **A3**. Y-axis scaling is adjusted for each movement type to emphasize fluorescence response differences. Data from the same trial are color coded. **B1-B3** Each panel shows data for a specific section of fast muscles, allowing comparison across muscle regions: **B1** Lateral, **B2** Intermediate, and **B3** Medial. Here, the Y-axis scaling is consistent across the panels, allowing direct comparison between muscle sections. Thick lines indicate the mean response, and thin lines represent ± SD (standard deviation)

To quantify the spatial activation patterns, we divided the fast muscle into three regions: lateral, intermediate, and medial (Fig. 3 A1, top). Fluorescence intensity was measured for each region. Figure 3 A2 displays the time course of fluorescence intensity for the lateral, intermediate, and medial regions. Upon escape, all the three regions exhibited clear increase in fluorescence intensity. The medial region showed the largest change (1.8 in ΔF/F0), while the lateral region showed the smallest change (0.4 in ΔF/F0). This trend was consistent across the population data (Fig. 4 A1). In all trials (n = 8 of eight fish), fluorescence intensity increased in all the three regions, with the largest changes (ΔF/F0 Max) in the medial region, and the smallest in the lateral region.

Figure 3 B1 illustrates a representative example for the turn movements. The turn occurred near the end of the scan in the second panel (t2) (indicated as “turn”). Given the duration of the turn movement was around 30 ms (Additional file 2), the “turn” extended into the next frame (t3), noting that each image comprises scan lines for ~ 100 ms. Upon the turn movement, a clear increase in fluorescent intensity was observed in the lateral region of the right muscle (Fig. 3 B1, t3). Quantification revealed that, in addition to the lateral region, the intermediate region also showed a slight increase in fluorescence intensity (Fig. 3 B2). In contrast, the medial region exhibited minimal changes in intensity. This pattern was consistent across the population data (Fig. 4 A2). In all trials (n = 8 of eight fish), significant increases in fluorescence intensity were noted in the lateral section. The intermediate region showed moderate increases, while the medial region exhibited very small increases (0.2–0.4) in three of eight trials. In the remaining five trials, changes in intensity were at the noise level (around 0.1).

Figure 3 C1 presents a representative example for the swimming. The second panel (t2) shows an image in which the fish performed swimming consistently throughout the scanning time, marked as “swimming”. In this image, an increase in fluorescence intensity was observed in the lateral regions on both sides of the muscles (Fig. 3 C1, t2). Figure 3 C2 illustrates the time course of fluorescence intensity, where two bouts of swimming occurred (denoted swimming at the bottom of the figure). During each swimming bout, an increase in fluorescence intensity was detected in the lateral region. In contrast, the intermediate and medial regions showed minimal changes in fluorescence intensity. This trend was consistent across the population data (Fig. 4 A3). In all trials (n = 8 of eight fish), fluorescence intensity increases were observed in the lateral sections, whereas intensity changes in the intermediate and medial regions remained at noise levels (approximately 0.1), suggesting that fast muscles in these regions were not recruited during slow swimming.

The above results describe only the spatial activation patterns of muscles for each type of movement, without addressing the amplitude of muscular activations across different movement types. To address this question, we plotted fluorescence intensity changes in each muscle region across three movement types (note that the vertical axis scales differ in Fig. 4 A1–A3, making it difficult to directly compare activation amplitudes across movements). As shown in Fig. 4 B1–B3, the amplitude of muscular activations was highest during escape movements across all three muscle regions. Notably, this was also the case for the lateral region, which, although showing the lowest activity among the three regions during escapes (Fig. 3 A2 and 4A1), still exhibited higher activation amplitudes than those observed during turns and swimming (Fig. 4B1).

In summary, calcium imaging experiments revealed distinct spatial recruitment patterns in fast muscles. During low-strength movements (slow swimming), only the lateral region was activated. As the fish engaged in stronger movements, more medially located muscles were recruited; during turns, both the intermediate and lateral regions became active, while during escapes all regions were activated. Additionally, within each region, the level of muscular activation tended to increase with the strength of the movement.

### Electrophysiological analyses of fast muscles during fictive swimming

In the calcium imaging experiments, rostral parts of the larval fish were embedded in agar. In this condition, fish only perform slow swimming with a maximum tail beat frequency (called swimming frequency hereafter) typically around 30 Hz [22]. In contrast, under unrestrained conditions, larval fish can swim with much higher swimming frequency, exceeding 65 Hz [14, 21–23]. Due to this technical limitation in the calcium imaging setup, it was not possible to examine fast muscle activation patterns during high-frequency swimming.

To circumvent this limitation, we conducted electrophysiological analyses of fast muscles during fictive swimming, which refers to neuronal or muscular activity associated with swimming in a paralyzed condition. In fictive swimming preparations, larval fish exhibit a broad range of swimming frequencies [14, 24]. For muscle activity recordings, we used *relaxed* mutants, whose muscles are immotile due to a defect in dihydropyridine receptors (DHPR) [25, 26]. Notably, defects in DHPR do not affect membrane voltage dynamics in muscles [27], enabling us to examine muscle electrical activity during fictive swimming.

We conducted electrophysiological experiments using 3-dpf larvae, as muscle cells in *relaxed* mutants begin to degenerate at 4 dpf. To investigate spatial activation patterns of muscles, we performed paired whole-cell patch-clamp recordings on two muscles located at different depths (superficial vs. more medial). Before describing the results of paired patch recordings, however, we first describe single recordings from fast muscle cells in the most superficial layer (layer 1 or L1; see Additional file 3 for the architecture of fast muscle in a 3-dpf larva) to illustrate how electrical activities of fast muscle electrical activity appear in electrophysiology traces during swimming.

**Fig. 5:**
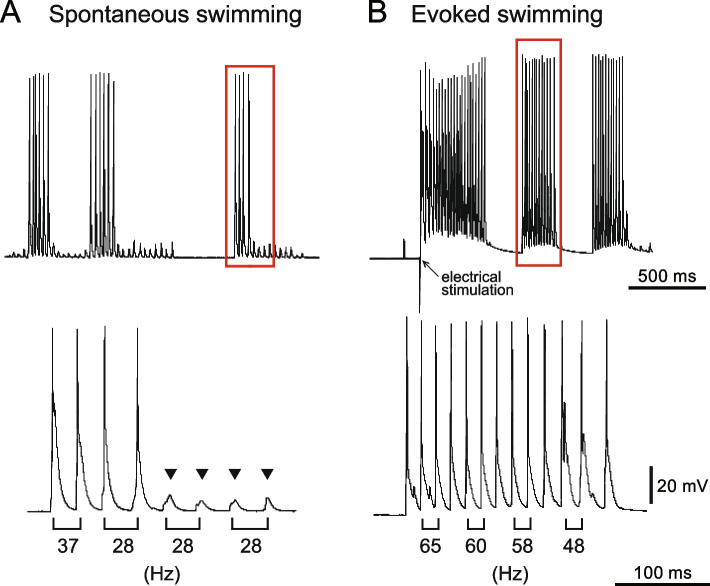
Electrophysiological recordings from an L1 (layer 1) fast muscle during fictive swimming. **A** Spontaneous swimming recording. The bottom panel provides a close-up view of the boxed area, highlighting individual swim cycles. Swimming frequencies for each cycle are labeled below the trace. Arrowheads mark subthreshold endplate potentials (EPPs), which represent signals that did not reach the threshold to trigger an action potential. **B** Evoked swimming recording. An electrical stimulus was applied at the point marked by the arrow. The bottom panel provides a close-up view of the boxed area, highlighting individual swim cycles

Figure 5 shows examples of such recordings. Without external stimulation, larvae occasionally engage in spontaneous swimming, characterized by periodic membrane voltage oscillation (Fig. 5A, top panel). In the close-up view (bottom panel), periodic action potentials (the first four sharp peaks) and subthreshold endplate potentials (EPPs; indicated as arrowheads) are evident. Electrically coupled potentials originating from neighboring fast muscle cells may contribute to these EPPs [28]. During the periods for which fast muscles exhibit subthreshold EPPs, fish continues swimming, as confirmed by paired recordings from fast muscles and slow muscles (Additional file 4, arrowheads); when fast muscles exhibit periodic EPP oscillations, slow muscles display clear electrical activities. Membrane voltage oscillation frequency, which corresponds to swimming frequency, tends to be higher at the beginning of swim bouts and gradually decreases as swimming continue. Correspondingly, fast muscle activity shifts from spiking to subthreshold EPPs. For instance, in the bottom panel of Fig. 5A, the muscle spikes during the first four cycles, then transitions to subthreshold EPP for the remaining cycles.

During spontaneous swimming, the swimming frequency typically ranges from 25 to 40 Hz. To induce higher swimming frequencies, we applied electrical stimulation, eliciting more vigorous swimming than spontaneous bouts (evoked swimming; compare muscle spiking activity in Fig. 5A and B), with frequencies reaching 40 to 70 Hz (Fig. 5B, bottom). By gathering data from both spontaneous and evoked swimming, we were able to analyze fast muscle recruitment patterns across a broad range of swimming frequencies.

**Fig. 6:**
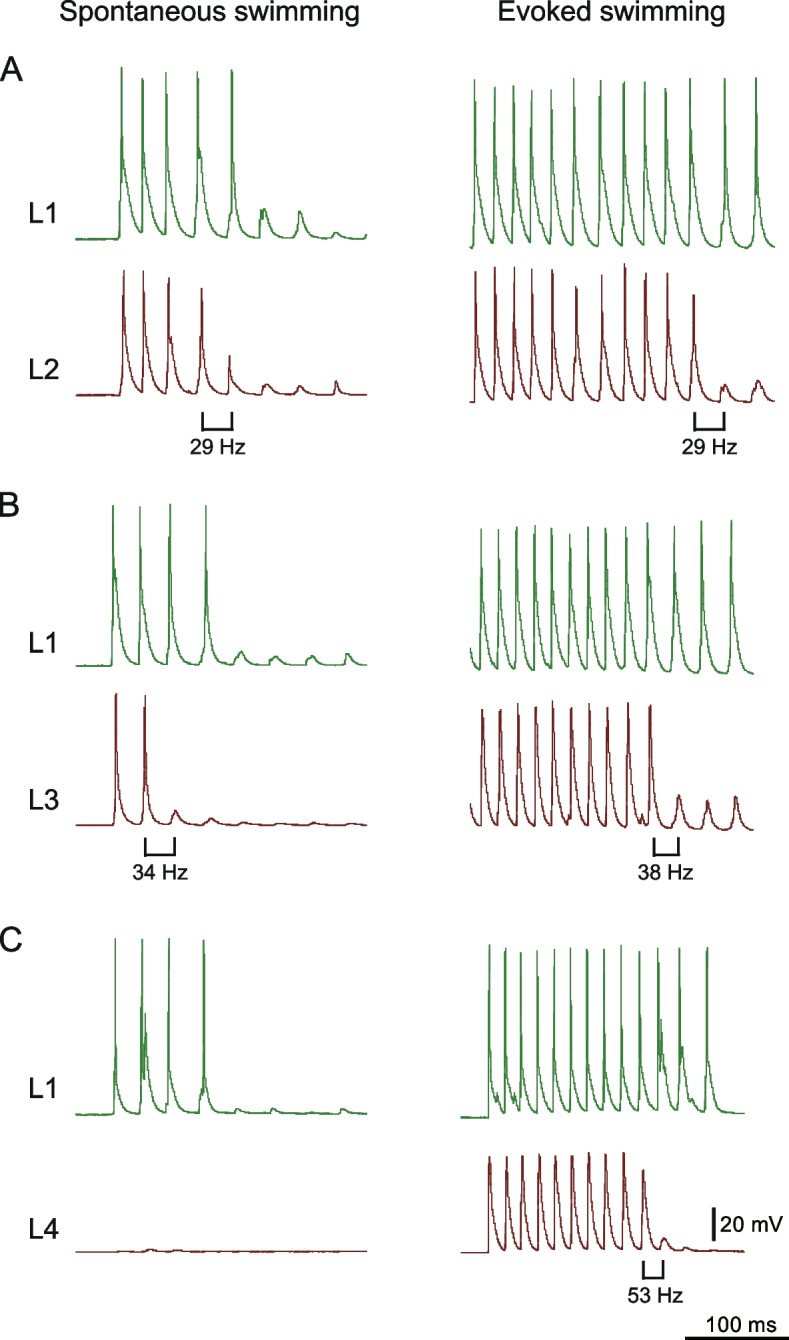
Paired electrophysiological recordings between L1 and deeper muscles (L2, L3, and L4). **A** Paired recording from L1 and L2 muscles. The left section presents a recording of L1 and L2 muscles during spontaneous fictive swimming, while the right section shows the same pair during evoked swimming. The swimming frequencies at which the transition from spikes to subthreshold EPPs occurred are indicated under the traces. **B** Same as **A**, but paired recording from L1 and L3. **C** Same as **A**, but paired recording from L1 and L4

Figure 6 shows representative examples of paired recordings from fast muscles in the superficial layer 1 (L1) and deeper layers (L2, L3, and L4). The left panels display recordings during spontaneous swimming, while the right panels show recordings during evoked swimming. Across all panels, a trend emerges: muscles in deeper layers (L2, L3, and L4) exhibited transitions from spiking to subthreshold EPPs earlier than the L1 muscles. For instance, in Fig. 6A (left panel), the L1 muscle exhibited spiking activities for the first five cycles, whereas the L2 muscle spiked for only the first four cycles. This suggests that, as swimming frequency gradually decreases, the L2 muscle de-recruits before the L1 muscle. Similar trends are observed in the paired recordings between L1 and L3 (Fig. 6B) and between L1 and L4 (Fig. 6C, right panel). Additionally, there appeared to be an orderly de-recruitment pattern, with deeper fast muscles de-recruiting at progressively higher swimming frequency (for comparison, the swimming frequency at which this transition occurred is noted below each panel). Notably, the L4 fast muscle was not recruited at all during spontaneous swimming (Fig. 6C, left panel). These results strongly suggest the presence of a sequential recruitment pattern among fast muscles, with orderly de-recruitment occurring as swimming frequency decreases.

**Fig. 7:**
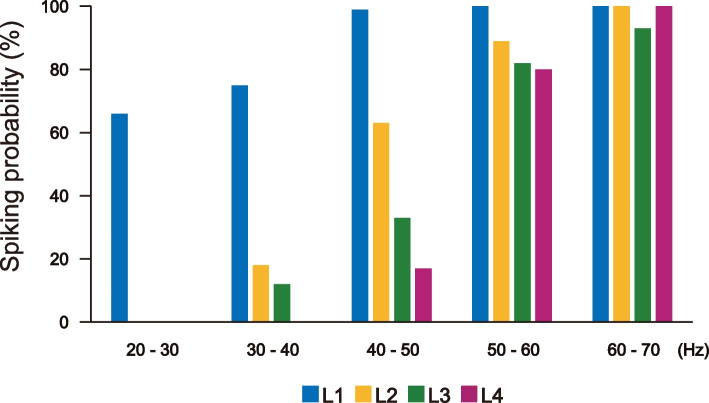
Relationship between swimming frequencies and probability of spiking events. This figure illustrates the relationship between swimming frequencies (X-axis) and the probability of spiking events in fast muscles. Data were gathered from nine paired recordings: three pairs of L1 vs. L2, L1 vs. L3, and L1 vs. L4

To quantitatively analyze recruitment patterns in fast muscles, we systematically examined the relationships between swimming frequency and the probability of spiking activities across each layer of muscles. Swimming frequencies were grouped into 10 Hz bins, and the probabilities of spiking at each frequency range was plotted for each layer of muscles (Fig. 7). Results from nine paired recordings (three for L1 vs. L2, three for L1 vs. L3, and three for L1 vs. L4) revealed clear patterns. At the lowest swimming frequency (20–30 Hz), only L1 muscles showed spiking activities (note that the Y-axis values in Fig. 7 do not represent the absolute recruitment ratio of L1 muscles at 20–30 Hz, as L1 muscles do not produce EPPs in every cycle; cycles without EPPs are excluded from analysis; see Additional file 4). In the 30–40 Hz bin, the probability of spiking activity per cycle in L1 muscles increased, and L2 and L3 muscles also showed spiking activities in some cycles. Importantly, the probability of spiking activity was highest in L1 muscles, followed by L2 and then L3, resulting in a downward slope across layers (Fig. 7). In the 40–50 Hz range, L4 muscles began to exhibit spiking activity, further reinforcing the downward-slope pattern. This trend continued in the 50–60 Hz range, with increased spiking activity for each muscle layer. At 60–70 Hz, muscles of all layers displayed spiking activities in nearly every cycle. These results clearly demonstrate an orderly recruitment pattern in fast muscles.

### Cross-sectional area analysis of fast muscle cells along the medio-lateral axis

**Fig. 8:**
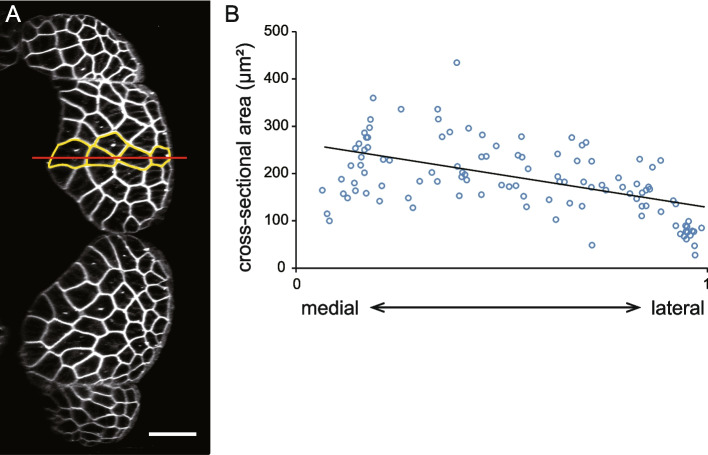
Size of fast muscles along medio-lateral axis. **A** Cross sectional fluorescence image of fast muscles in a 5-dpf Tg(*actc1b*:Ace2N-4aa-mNeonGreen), showing membrane-tethered mNeonGreen expression specifically in fast muscles. Only the right muscles are shown. Fast muscle cells intersecting the red line (highlighted in yellow) were selected for analysis. Scale bar, 30 µm. **A** Cross sectional area of fast muscles. The medial edge of the fast muscle cell mass is assigned position value 0, while the lateral edge is assigned position value 1 (X-axis). Data were obtained from 12–14 muscle segments per fish across five fish, totaling 15 analyzed segments

According to the size principle, faster/stronger motor units tend to have larger motoneurons and muscle fibers [8, 9]. Based on this principle, and considering the results from calcium imaging and electrophysiology, we hypothesized that fast muscle fiber size might increase medially along the medio-lateral axis. To test this, we examined the cross-sectional area of fast muscle cells at the dorso-ventral depth level of the central canal in 5-dpf larvae (Fig. 8A), corresponding to the optical sections used in calcium imaging experiments (Figs. 2 and 3). Analysis of 15 muscle segments (three segments were examined in each of five fish) revealed the expected trend: fast muscles in more medial regions were generally larger than those in lateral regions (Fig. 8B).

## Discussion

### Methodological considerations

We used calcium imaging and electrophysiology to study spatial activation patterns of fast muscles during behaviors of various intensities. Each method offers distinct advantages and limitations. The advantages of calcium imaging are as follows. First, imaging can be performed on larvae exhibiting actual movements, excluding any side effect associated with paralysis. Second, calcium imaging allows us to directly observe spatial activation patterns of muscles in a wide area of muscle tissue. Third, several movement types can be evoked, and analyzed. However, calcium imaging also has several limitations. First, time resolution is low. For instance, once a very strong movement such as an escape occurs, the calcium signal persists for a second or more, lasting well beyond the muscular contractions. Second, fish do not engage in fast swimming when embedded in agar [22], preventing us from capturing calcium imaging data during high-frequency swimming. Moreover, even if larvae could perform fast swimming under such conditions, analyzing muscle activation patterns on a cycle-by-cycle basis would remain challenging due to the poor temporal resolution; the calcium signal from a strong movement could dominate the signal series, obscuring subsequent signals. For example, if a fast swimming event follows an escape response (commonly observed in unrestrained fish), the calcium signal from the escape might overshadow that from fast swimming. Therefore, in practical terms, calcium imaging is best suited to studying activation patterns in single-body movements (e.g., escape and turn) or in behaviors with relatively steady force, such as slow swimming.

Electrophysiology offers several advantages. First, it provides exceptional temporal resolution, enabling precise, cycle-by-cycle analyses during swimming across a broad range of frequencies [14]. Second, fictive swimming with various frequencies can be reliably evoked, allowing a comprehensive analysis of activation patterns over different swimming intensities. However, electrophysiology also has limitations. First, fish must be paralyzed for these recordings. While fictive movement studies often use neuromuscular blockers such as α-bungarotoxin or tubocurarine to study neural activity [18, 29], this approach was unsuitable here because these blockers prevent muscle activation. This required us to use *relaxed* mutants, which led to some restrictions, including the need to use younger larvae (3 dpf) due to health issues in older mutant larvae. At this stage, zebrafish larvae do not consistently exhibit visually or vestibularly evoked behaviors, thereby limiting the behaviors available for analysis. Another limitation is that only a limited number of cells can be recorded during a single session. In this study, for instance, recordings were limited to cell pairs per session, making it necessary to pool data across multiple recordings to assess spatial activation patterns (Fig. 7). Additionally, recording from deeper muscle cells poses technical challenges, as the muscle layers above each target cell must be carefully removed. Consequently, the deepest layer analyzed in this study was L4, leaving more medial fast muscle regions otuside the scope of our analysis (Additional file 3).

The strengths and limitations of calcium imaging and electrophysiology are, in many respects, complementary. Electrophysiology offers high temporal resolution, while calcium imaging provides broad spatial coverage across muscle tissue. Additionally, calcium imaging allows recordings in freely moving larvae, avoiding potential side effects of paralysis necessary in electrophysiology. Importantly, both methods yielded consistent and complementary results, reinforcing our main conclusion that fast muscle recruitment progresses from lateral to medial regions as movement speed or strength increases.

### Strategies for increasing muscular contraction power

To enhance muscular contraction power for producing high-speed and/or strong movements, vertebrates employ two primary strategies. One approach is to recruit additional motor units that remain inactive during low power movements. The second approach involves increasing the activity levels of motor units that are already engaged. It is known that mammals use both strategies [7]. In this study, we focused specifically on spatial recruitment patterns of fast muscles, without extensive analysis of the second strategy. However, this does not mean that fish lack the ability to modulate the activity levels of active motor units. Our calcium imaging experiments demonstrated that escape movements elicited the highest calcium responses across all fast muscle regions (Fig. 4 B1–B3). Since an escape movement involves a single rapid body bend, this finding suggests that fast muscles likely generate multiple spikes during each escape bend. This idea of multiple spikes per single bend during high-intensity movements is further supported by our electrophysiology data. We observed that fast muscles often produced multiple spikes per cycle during fictive swimming immediately following electrical stimulation (Additional file 5). Thus, similar to mammals, zebrafish likely employ both strategies—recruiting additional motor units and increasing firing frequencies of active units—to boost muscular contraction power for high-speed and/or powerful movements.

### Physiological significance of spatially-ordered recruitments

The present study on zebrafish larvae identifies a spatially-ordered recruitment pattern in fast muscles, where muscle activation progresses from lateral to medial regions as movement strength increases. This organization continuously extends to the most superficial slow muscles. In mammals, such a spatially ordered recruitment pattern is absent, as slow and fast muscles, including their subtypes, are intermingled. Below, we explore the physiological significance of this medio-lateral recruitment pattern and possible reasons fish may adopt such an arrangement.

The distinct muscular arrangement in fish versus mammals likely reflects differences in force transmission systems. In mammals, muscle contractions primarily change joint angles between two bones, and typical muscles (such as parallel muscles like the biceps) connect to bones via small-diameter tendons. In this setup, there appears to be little mechanistic advantage to contracting confined muscle areas, since the force ultimately converges through small tendon areas. In pennate muscles, tendons are broader; however, the main reason for taking the pennate shape is that it is thought to house more muscle fibers for greater force production, and not to change shapes of tendons or bones. Thus, the force transmission system is not fundamentally different from that of parallel muscles.

By contrast, fish use a different approach. Muscle contractions primarily bend the body, which includes the large muscle mass, spinal cord, and notochord (or spine in adults). Muscle segments attach to a collagen-rich, sheet-like connective tissue called the myoseptum, which spans large attachment areas between muscle segments. The medial side of the myoseptum anchors to the spinal cord and notochord (or spines in adult fish), while its lateral edge connects to the skin. This configuration enables a highly complex, three-dimensional force transmission system for a wave of muscle activation from head to tail to produce a smooth, caudally-moving bend along the body during swimming. Given that the sheet-like myoseptum plays a crucial role, contractions in different muscle regions likely result in different effects on body movement. The superficial location of slow muscles in nearly all fish suggests mechanistic or energetic advantages, which our findings indicate may extend throughout the fast muscle mass. If we consider force transmission as a lever-like system, contractions of laterally located muscles—due to their greater distance from the body midline—produce larger moments of force. The lateral-to-medial recruitment order then may provide finely-tuned, energy-efficient body bending. This may also explain the muscle size gradient; smaller, more laterally positioned fast muscles may enable finer control, while larger, medial fast muscles provide greater force.

Could fast muscles in zebrafish larva be subdivided into specialized types, akin to the FF and FR muscle types seen in mammals? Current data do not support this, as there are no genetic markers identifying distinct fast muscle regions. Furthermore, the continuous recruitment pattern and size gradient along the medio-lateral axis (Figs. 7 and 8) suggest a continuum of muscle properties rather than distinct subtypes. Thus, we favor the idea that muscle characteristics shift continuously along this axis.

Are these spatial recruitment and size gradients of fast muscles in zebrafish larvae relevant to adults and other fish? Regarding spatially-ordered recruitment, a previous study in adult rainbow trout by Ellerby and Altringham [12] demonstrated that superficially located fast muscles are active during fast swimming, while those in the most medial region are inactive during this activity, becoming active only during escape responses. Given this finding, we expect that spatially-ordered recruitment could be a general strategy across fish species. However, the size gradient in fast muscles remains unreported in adults. Notably, many adult fish, including zebrafish, develop intermediate muscle fibers that combine slow and fast characteristics and have intermediate sizes [30]. Consequently, a gradient in muscle size (fast > intermediate > slow) along the medio-lateral axis is present in adult fish even if size gradient is absent among fast muscles. Additionally, if there is an upper size limit for muscle fibers due to metabolic or other physiological constraints, fast muscle fibers in adult fish may reach to this upper limit, resulting in a more uniform size distribution. Further research is needed to clarify these aspects in adult fish.

## Conclusions

In this study, we investigated the activation patterns of fast muscles in larval zebrafish during movements of varying speeds and strengths, employing both calcium imaging and electrophysiology. Our findings from these independent methods converge on a consistent conclusion: there exists a spatially-ordered recruitment pattern among fast muscle cells. Specifically, during weaker or slower movements, only the lateral portions of fast muscle cells are activated. As movement speed or strength increases, we observe a progressive recruitment of additional fast muscle cells, following a clear spatial order from lateral to medial regions. Additionally, our anatomical studies revealed that muscle fiber size increases systematically from lateral to medial. Thus, the spatially-ordered recruitment of fast muscles from lateral to medial is associated with a corresponding increase in muscle fiber size. These findings provide significant insights into the organization and function of fast muscles in larval zebrafish, illustrating how spatial recruitment and fiber size interact to optimize movement performance.

## Methods

### Fish care and strains

Zebrafish adults, embryos, and larvae were maintained at 28.5℃. Animals were staged according to days post fertilization (dpf). Tg(*smyhc1*:tdTomato)nns101 and Tg(*actc1b*:Ace2N-4aa-mNeonGreen)nns102 were generated using the Tol2-mediated transgenesis, with the *smyhc1* promoter [31] for the former and the *actc1b* (formally, called *α-actin*) promoter [32] and the Ace2N-4aa-mNeon reporter [33] for the latter. Tg(*actc1b*:GFP)zf13 (formally, called Tg(*α-actin*:GFP)) was described in Higashijima et al. [32]. For calcium imaging, we used Tg(*actc1b*:tdTomato-jGCaMP7f)nns103 [34]; formally, Tg(*α-actin*:tdTomato-jGCaMP7f)), in which tdTomato-jGCaMP7f fusion protein are expressed in fast muscles. In the text, this line is referred to as Tg(*actc1b*:jGCaMP7f) since we utilized only the green fluorescence signal derived from jGCaMP7f for calcium imaging, not the tdTomato signal. Confocal imaging both for morphological observation and functional calcium imaging was performed using *nacre* mutants which lack black pigment cells. The immotile mutant fish *relaxed* (*red*^*mi90*^ [26]) was used for electrophysiological studies.

### Morphological observation of slow and fast muscles and muscle size measurement

Compound transgenic fish lines, either Tg(*actc1b*:GFP); Tg(*smyhc1*:tdTomato) or Tg(*actc1b*:Ace2N-4aa-mNeonGreen); Tg(*smyhc1*:tdTomato) were laterally mounted in low-melting point agarose (1.5%; Thermo Fisher Scientific) in glass-bottomed 35-mm plastic dishes. Confocal imaging was performed with an SP8 Leica microscope equipped with a 40 × water immersion objective (NA,1.1) and a 488 nm laser. Green (GFP or mNeonGreen) and red (tdTomato) fluorescence were separated by a prism, and detected by HyD detectors. Cross sectional views were generated from confocal images using Imaris (Bitplane) software.

Muscle size measurement were conducted on fast muscles using fluorescence images of Tg(*actc1b*:Ace2N-4aa-mNeonGreen). Individual cell membranes were manually traced in ImageJ/Fiji software to determine muscle size. In Fig. 8, only the green channel (Ace2N-4aa-mNeonGreen signals) is shown.

### Calcium imaging of fast muscles during escapes, turns, and forward swimming

The setup for eliciting escape behaviors has been previously described [17–19]. Briefly, we used a custom sound/vibration stimulation system with an audio speaker attached to an acrylic plate. The stimulation signal (500 Hz, two cycles) was delivered to the speaker. A screen was placed below the acrylic plate such that we could deliver visual stimuli from below (Fig. 2; Additional file 1). To induce turns and forward swimming, we used moving grating stimuli as described by Orger et al. [20]. To prevent interference with calcium imaging via GCaMP’s green fluorescence signal, a long pass filter (Olympus, BA580IF) was attached to the exit light path of the projector (Philips). Command for producing moving gratings in the projector was generated by a computer with a custom-written software in C ++ with OpenGL library.

Five-day-post-fertilization (5 dpf) larvae of Tg(*actc1b*:jGCaMP7f) line were mounted upright in 1.5% of low-melting point agarose in glass-bottomed 35-mm plastic dishes. Agarose covering the tail part was removed to allow tail movement. Cutting position was set at muscle segment 18 or 19. The dish was then attached to the acrylic plate. This setup was positioned on a FV1200 confocal upright microscope (Olympus). Tail movements were monitored from below using a setting described in Additional file 1 with infrared LED illumination and recorded at 1,000 frames/second using a high-speed camera (acA640-750um, Basler). The size of the small mirror for deflecting LED illumination was 4 × 5 mm.

To measure angular velocity of the tail movements, we recorded tail flexion angles between two positions along the midline: position #1 at the edge of the agar (muscle segment 18 or 19) and position #2 at the 30% of the free tail length (Additional file 2). The angle was calculated between the line through these two positions at maximum bend and the body line prior to movement. Determination of the position #2 at the time frame of the maximum bend was aided by xanthophore pigmentation visible with infrared illumination. For measuring angular velocity in the middle of the swimming, similar analyses were conducted except that the time 0 was set when the position #2 passed the midline.

Calcium imaging was conducted with an FV1200 confocal microscope using a 16 × water immersion objective (CFI75 LWD 16X W, NA0.8, Nikon) and a 473 nm laser. A 490–540 nm band-pass filter served as an emission filter. We focused on fast muscles in segments 12–14, setting the focal plane at the central canal of the spinal cord. Sequential images (128 × 48 pixels) were captured over 12–33 s (150–400 frames) at 10 frames per second with a fully open pinhole. A trigger signal, generated by the confocal scanner, was used to synchronize confocal images and high-speed camera images. The same trigger signal was used to deliver a time-locked sound/vibration stimulation to the larvae: the timing of the stimulation was set such that escape movements occurred when the scan line was moving at the upper image region (see Fig. 3 A1). For escape or turn movements, we only analyzed those trials in which only single tail bend occurred.

### Analysis of calcium imaging data

The left and right edges of the fast muscles were manually determined by visual inspections. Vertical lines were drawn from the top to the bottom to delineate region of interest. The boxed area was then divided vertically into nine sections using the ImageJ/Fiji. The middle three sections (area 4, 5, and 6 from the left) represented the spinal cord and were excluded from analyses. The remaining sections were designated as fast muscle region, resulting in three subdivisions on each side: lateral, intermediate, and medial. Before the quantification of fluorescence intensity, background subtraction was performed using the mean pixel intensity from an area outside the sample. The mean pixel intensity of each fast muscle section was then calculated. All the analyses were conducted using ImageJ/Fiji.

### Electrophysiology

Whole-cell recordings from muscles were performed using 3-dpf larvae that were homozygous for *relaxed*. The *relaxed* gene encoded dihydropyridine receptors (DHPR) [26], which interact directly with ryanodine receptors to release Ca^2+^ from the sarcoplasmic reticulum (SR) into the cytosol. In homozygous *relaxed* mutants, this Ca^2+^ release mechanism is defective, resulting in complete paralysis [25]. Importantly, DHPR functional defects do not affect membrane voltage dynamics in muscle cells [27].

Patch-clamp electrophysiological recordings were conducted as described previously [24, 35, 36], with some modifications. Larvae were secured by pinning through the notochord to a Sylgard-coated glass-bottomed dish using short fine tungsten pins, then submerged in extracellular recording solution containing (in mM): 134 NaCl, 2.9 KCl, 1.2 MgCl_2_, 2.1 CaCl_2_, 10 HEPES, and 10 glucose, adjusted to pH 7.8 with NaOH. The skin around muscle segments 15–19 was removed with forceps. The preparations were observed using a water immersion objective (40 × ; NA 0.80; Olympus) on an upright microscope (BX51WI; Olympus) with differential interference contrast optics. The recording sites were established as the follows: at the dorso-ventral level, the sites were positioned around the ventral edge of the notochord. At the rostro-caudal level, the first recording site, which targeted superficially located muscles, was positioned at muscle segment 16. The second recording site, intended for more medially located muscles, was set two segments caudal to the first. For recordings of L1 fast muscle cells, the overlying slow muscles were carefully removed with glass pipettes. Similarly, for recordings of deeper-layer fast muscles, both slow and fast muscles covering the target fast muscles were peeled away. For example, to access the L4 fast muscles, the overlying slow muscles and the L1, L2, and L3 fast muscles were removed.

Whole-cell recordings were acquired using MultiClamp 700B amplifiers and digitized with Digidata 1390 (Molecular Devices). Patch electrodes (resistance, 5–10 MΩ were filled with the intracellular solution containing (in mM): 119 K-gluconate, 6 KCl, 2 MgCl_2_, 10 HEPES, 10 EGTA, and 4 Na_2_ ATP at 290 mOsm, adjusted to pH 7.2 with KOH. Fictive swimming with low-swimming frequency occurred spontaneously. To induce high frequency fictive swimming, a brief electric shock (7–20 V for 0.2–1.0 ms) were occasionally applied to the tail.

### Analysis of electrophysiology data

Electrophysiological data were analyzed with DataView (software by William Heitler, University of St. Andrews) and Microsoft Excel. Spiking activity of recorded fast muscles was defined by voltage elevation events that exceeded 70% of the peak elevation (mean of the top ten events). To determine the frequency of swimming, the duration between a spike (or end plate potential, EPP) of the analysis target and the subsequent spike (or EPP) of the next cycle was defined as the cycle period. Swimming frequency was then calculated as the inverse of the cycle period. For each spike (or EPP), the 70% rise time point from the trough to the peak was recorded as the timing of the spike (or EPP).

A total of nine paired recordings were conducted between fast muscles (three for L1 vs. L2, three for L1 vs. L3, and three for L1 vs. L4). In addition, three paired recording between slow muscles and L1 fast muscles were conducted. Data from each paired recording were stored approximately every 30 s. Within each file (consisting of ~ 30 s of recording), one electrical stimulation was applied. To examine the relationship between swimming frequency and the probability of spiking activity (Fig. 7), at least 40 cycles were analyzed from each paired recording using the following method. For spontaneous swimming, cycles from the first swim bout containing more than 10 consecutive cycles with at least five spiking activities were analyzed. For evoked swimming, cycles from the second swim bout occurring after electrical stimulation were analyzed. This procedure was repeated across files until the total number of analyzed cycles exceeded 40 for each paired recording. Data for frequencies of 20–30 Hz and 30–40 Hz were derived from spontaneous swimming, while data for 40–50 Hz were collected from both spontaneous and evoked swimming, with half from each type. Data for frequencies of 50–60 Hz and 60–70 Hz were exclusively from evoked swimming.

## Supplementary Information

Supplementary Material 1

Supplementary Material 2

Supplementary Material 3

Supplementary Material 4

Supplementary Material 5

## Data Availability

Datasets used and/or analyzed during this study are available from the corresponding author on request.

## References

[1] Hess A. Vertebrate slow muscle fibers. Physiol Rev. 1970;50(1):40–62. 4904270 10.1152/physrev.1970.50.1.40

[2] Luna VM, Daikoku E, Ono F. “Slow” skeletal muscles across vertebrate species. Cell Biosci. 2015;5:62. 26568818 10.1186/s13578-015-0054-6 PMC4644285

[3] Schiaffino S, Reggiani C. Fiber types in mammalian skeletal muscles. Physiol Rev. 2011;91(4):1447–531. 22013216 10.1152/physrev.00031.2010

[4] Eccles JC, Eccles RM, Lundberg A. The action potentials of the alpha motoneurones supplying fast and slow muscles. J Physiol. 1958;142(2):275–91. 13564435 10.1113/jphysiol.1958.sp006015 PMC1356679

[5] Fetcho JR. The spinal motor system in early vertebrates and some of its evolutionary changes. Brain Behav Evol. 1992;40(2–3):82–97. 1422809 10.1159/000113905

[6] Heckman CJ, Enoka RM. Motor unit. Compr Physiol. 2012;2(4):2629–82. 23720261 10.1002/cphy.c100087

[7] Burke RE. Motor units: anatomy, physiology and functional organization. In: Handbook of Physiology. Section I. The Nervous System. Vol. II Motor Systems, V.B. Brooks, Editor. Washington: American Physiological Society. 1981;354–422.

[8] Henneman E, Mendell LM. Functional organization of motoneuron pool and its inputs. In: Handbook of Physiology. Section 1: The Nervous System. Vol. II Motor Control, Part 1, V.B. Brooks, Editor. Bethesda: American Physiological Society. p. 1981;423–507.

[9] Zajac FE, Faden JS. Relationship among recruitment order, axonal conduction velocity, and muscle-unit properties of type-identified motor units in cat plantaris muscle. J Neurophysiol. 1985;53(5):1303–22. 2987433 10.1152/jn.1985.53.5.1303

[10] Jayne BC, Lauder GV. How swimming fish use slow and fast muscle fibers: implications for models of vertebrate muscle recruitment. J Compar Physiol a-Sensory Neural Behavior Physiol. 1994;175(1):123–31. 10.1007/BF00217443 8083846

[11] Tsukamoto K. Contribution of the red and white muscles to the power output required for swimming by the yellowtail. Bull Jpn Soc Sci Fish. 1984;50(12):2031–42.

[12] Ellerby DJ, Altringham JD. Spatial variation in fast muscle function of the rainbow trout Oncorhynchus mykiss during fast-starts and sprinting. J Exp Biol. 2001;204(Pt 13):2239–50. 11507108 10.1242/jeb.204.13.2239

[13] Bello-Rojas S, et al. Central and peripheral innervation patterns of defined axial motor units in larval zebrafish. J Comp Neurol. 2019;527(15):2557–72. 30919953 10.1002/cne.24689 PMC6688944

[14] McLean DL, et al. Continuous shifts in the active set of spinal interneurons during changes in locomotor speed. Nat Neurosci. 2008;11(12):1419–29. 18997790 10.1038/nn.2225 PMC2735137

[15] Menelaou E, McLean DL. A gradient in endogenous rhythmicity and oscillatory drive matches recruitment order in an axial motor pool. J Neurosci. 2012;32(32):10925–39. 22875927 10.1523/JNEUROSCI.1809-12.2012 PMC3428065

[16] Dana H, et al. High-performance calcium sensors for imaging activity in neuronal populations and microcompartments. Nat Methods. 2019;16(7):649–57. 31209382 10.1038/s41592-019-0435-6

[17] Kohashi T, Oda Y. Initiation of Mauthner- or non-Mauthner-mediated fast escape evoked by different modes of sensory input. J Neurosci. 2008;28(42):10641–53. 18923040 10.1523/JNEUROSCI.1435-08.2008 PMC6671347

[18] Satou C, et al. Functional role of a specialized class of spinal commissural inhibitory neurons during fast escapes in zebrafish. J Neurosci. 2009;29(21):6780–93. 19474306 10.1523/JNEUROSCI.0801-09.2009 PMC6665578

[19] Shimazaki T, et al. Behavioral role of the reciprocal inhibition between a pair of Mauthner cells during fast escapes in zebrafish. J Neurosci. 2019;39(7):1182–94. 30578342 10.1523/JNEUROSCI.1964-18.2018 PMC6381243

[20] Orger MB, et al. Control of visually guided behavior by distinct populations of spinal projection neurons. Nat Neurosci. 2008;11(3):327–33. 18264094 10.1038/nn2048 PMC2894808

[21] Budick SA, O’Malley DM. Locomotor repertoire of the larval zebrafish: swimming, turning and prey capture. J Exp Biol. 2000;203(Pt 17):2565–79. 10934000 10.1242/jeb.203.17.2565

[22] Severi KE, et al. Neural control and modulation of swimming speed in the larval zebrafish. Neuron. 2014;83(3):692–707. 25066084 10.1016/j.neuron.2014.06.032 PMC4126853

[23] Burgess HA, Granato M. Sensorimotor gating in larval zebrafish. J Neurosci. 2007;27(18):4984–94. 17475807 10.1523/JNEUROSCI.0615-07.2007 PMC6672105

[24] Kimura Y, Higashijima S-I. Regulation of locomotor speed and selection of active sets of neurons by V1 neurons. Nat Commun. 2019;10(1):2268. 31118414 10.1038/s41467-019-09871-x PMC6531463

[25] Granato M, et al. Genes controlling and mediating locomotion behavior of the zebrafish embryo and larva. Development. 1996;123:399–413. 9007258 10.1242/dev.123.1.399

[26] Zhou W, et al. Non-sense mutations in the dihydropyridine receptor beta1 gene, CACNB1, paralyze zebrafish relaxed mutants. Cell Calcium. 2006;39(3):227–36. 16368137 10.1016/j.ceca.2005.10.015

[27] Ono F, et al. Paralytic zebrafish lacking acetylcholine receptors fail to localize rapsyn clusters to the synapse. J Neurosci. 2001;21(15):5439–48. 11466415 10.1523/JNEUROSCI.21-15-05439.2001 PMC6762670

[28] Luna VM, Brehm P. An electrically coupled network of skeletal muscle in zebrafish distributes synaptic current. J Gen Physiol. 2006;128(1):89–102. 16801383 10.1085/jgp.200609501 PMC2151551

[29] Kimura Y, Okamura Y, Higashijima S. alx, a zebrafish homolog of Chx10, marks ipsilateral descending excitatory interneurons that participate in the regulation of spinal locomotor circuits. J Neurosci. 2006;26(21):5684–97. 16723525 10.1523/JNEUROSCI.4993-05.2006 PMC6675258

[30] Mascarello F, Romanello MG, Scapolo PA. Histochemical and immunohistochemical profile of pink muscle fibres in some teleosts. Histochemistry. 1986;84(3):251–5. 2940205 10.1007/BF00495791

[31] Elworthy S, et al. Expression of multiple slow myosin heavy chain genes reveals a diversity of zebrafish slow twitch muscle fibres with differing requirements for Hedgehog and Prdm1 activity. Development. 2008;135(12):2115–26. 18480160 10.1242/dev.015719

[32] Higashijima S, et al. High-frequency generation of transgenic zebrafish which reliably express GFP in whole muscles or the whole body by using promoters of zebrafish origin. Dev Biol. 1997;192(2):289–99. 9441668 10.1006/dbio.1997.8779

[33] Gong Y, et al. High-speed recording of neural spikes in awake mice and flies with a fluorescent voltage sensor. Science. 2015;350(6266):1361–6. 26586188 10.1126/science.aab0810 PMC4904846

[34] Sugioka T, Tanimoto M, Higashijima SI. Biomechanics and neural circuits for vestibular-induced fine postural control in larval zebrafish. Nat Commun. 2023;14(1):1217. 36898983 10.1038/s41467-023-36682-y PMC10006170

[35] Buss RR, Drapeau P. Physiological properties of zebrafish embryonic red and white muscle fibers during early development. J Neurophysiol. 2000;84(3):1545–57. 10980026 10.1152/jn.2000.84.3.1545

[36] Hirata H, et al. accordion, a zebrafish behavioral mutant, has a muscle relaxation defect due to a mutation in the ATPase Ca2+ pump SERCA1. Development. 2004;131(21):5457–68. 15469975 10.1242/dev.01410

