# Supplementary material for: Spatially ordered recruitment of fast muscles in accordance with movement strengths in larval zebrafish
Source: Zoological Lett. 2025 Jan 3;11:1. doi: 10.1186/s40851-024-00247-8 (PMC11697752; doi:10.1186/s40851-024-00247-8)
Supplement: Supplementary file 1 — Supplementary Material 1 [file 40851_2024_247_MOESM1_ESM.pdf]

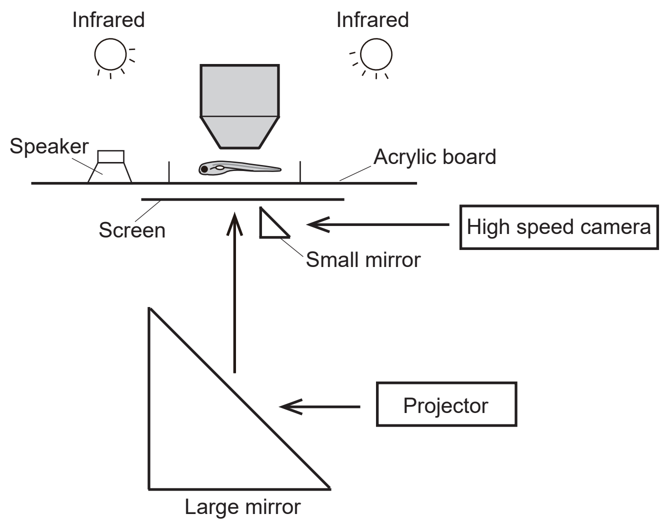

Additional File 1
Imaging setup schematic
The larval fish is illuminated by infrared lights, allowing the high-speed camera to capture the silhouette of the tail. A small mirror is placed in the light path to reflects the infrared lights. Moving gratings generated by the projector are reflected by a large mirror and projected onto a screen beneath the fish.
