# Supplementary material for: Spatially ordered recruitment of fast muscles in accordance with movement strengths in larval zebrafish
Source: Zoological Lett. 2025 Jan 3;11:1. doi: 10.1186/s40851-024-00247-8 (PMC11697752; doi:10.1186/s40851-024-00247-8)
Supplement: Supplementary file 2 — Supplementary Material 2 [file 40851_2024_247_MOESM2_ESM.pdf]

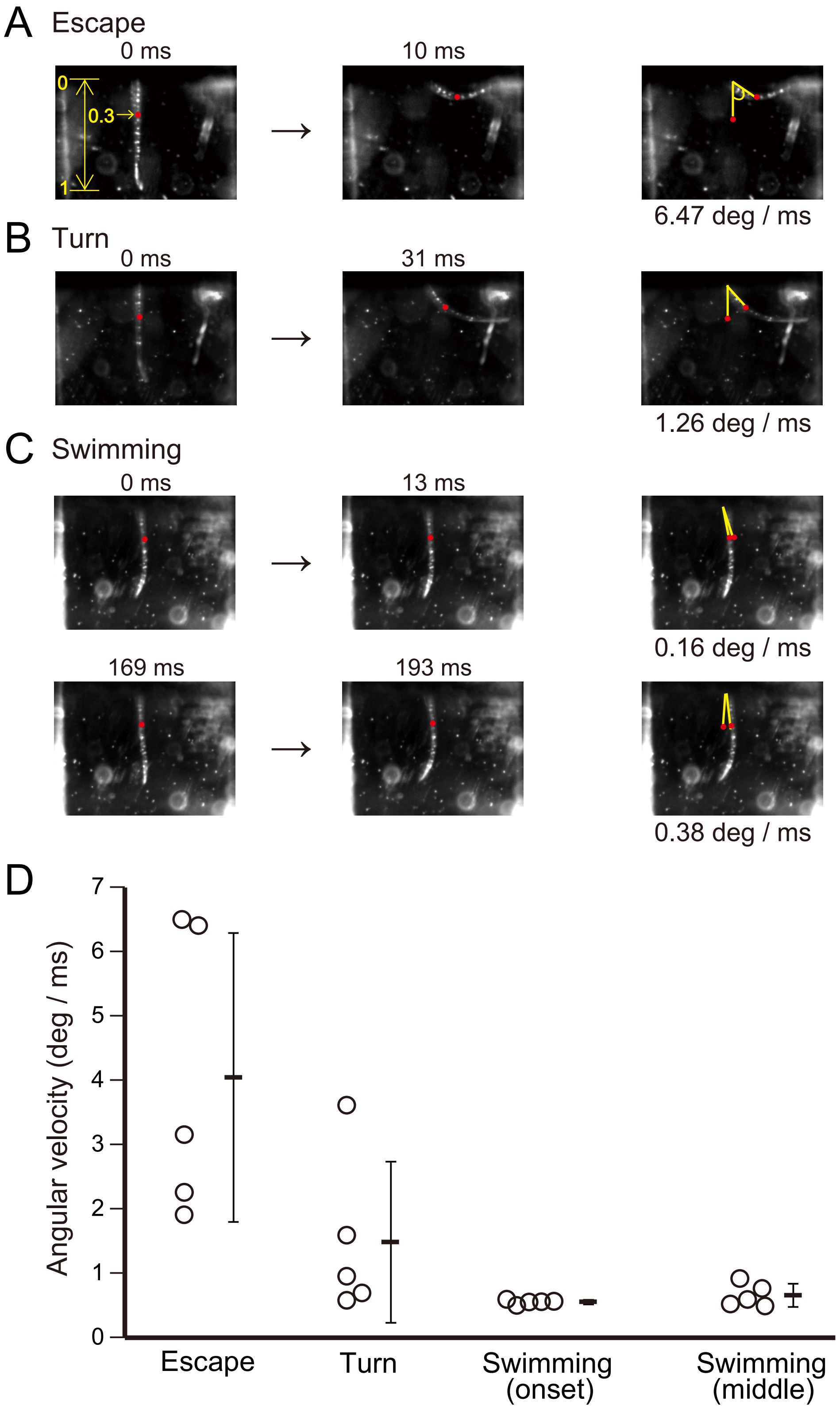

Additional File 2

Movements of larval fish in response to vibration and visual stimuli

A An example of escape behavior triggered by vibration stimuli. The left panel shows the initial position right before the movement onset (0 ms). The endpoint of the agarose is set as position 0, and the tail tip as position 1, with a reference point at 0.3 (red dot) for calculating angular velocity. The middle panel shows the frame with the largest reference point shift. The right panel presents the calculated angular velocity.

B An example of turn behaviors evoked by laterally-moving gratings.

C An example of swimming behaviors triggered by forwardly-moving gratings. The top row shows images at the swimming onset, and the bottom row shows images during mid-swimming.

D Graphs illustrating the angular velocities for escape, turn, and swimming trials. Thick and thin lines represent the mean and ±SD (standard deviation), respectively.
