# Supplementary material for: Spatially ordered recruitment of fast muscles in accordance with movement strengths in larval zebrafish
Source: Zoological Lett. 2025 Jan 3;11:1. doi: 10.1186/s40851-024-00247-8 (PMC11697752; doi:10.1186/s40851-024-00247-8)
Supplement: Supplementary file 3 — Supplementary Material 3 [file 40851_2024_247_MOESM3_ESM.pdf]

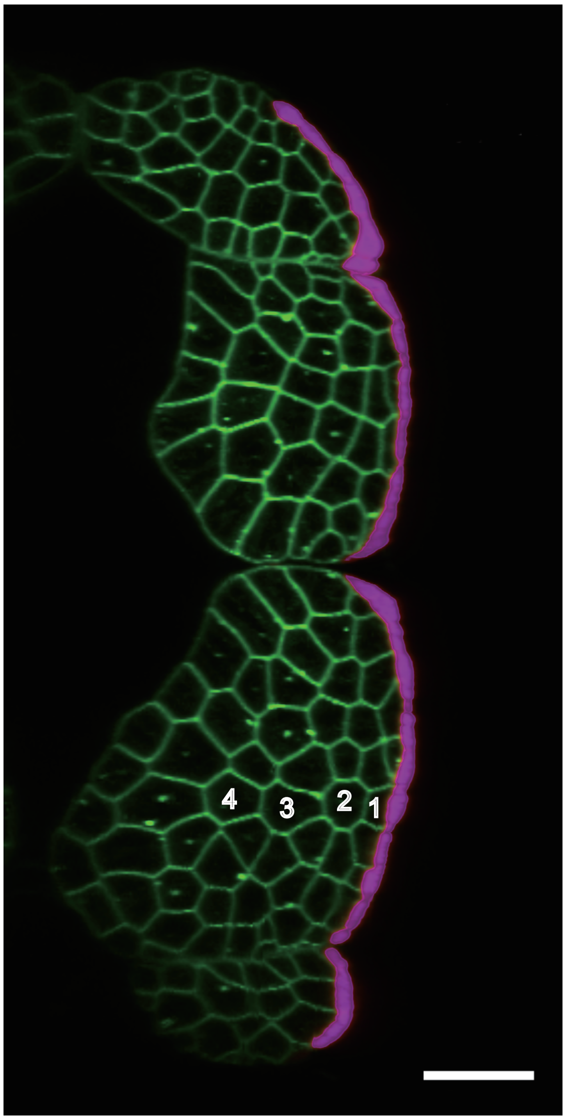

Additional File 3

Cross sectional view of fast and slow muscles in a 3-dpf larva

This image is a composite generated from confocal images of a Tg (actc1b:Ace2n-4aa-mNeonGreen); Tg(smyhc1:tdTomato) transgenic fish at 3 dpf. In this fish, membrane-tethered mNeonGreen (green signal) is expressed in fast muscles and tdTomato (magenta signal) in slow muscles. Numbers (1, 2, 3, and 4) indicate different depth levels of fast muscle layer. These correspond to the regions where electrophysiological recordings were performed on L1, L2, L3, and L4 fast muscles. Scale bar, 30 μm.
