# Supplementary material for: Spatially ordered recruitment of fast muscles in accordance with movement strengths in larval zebrafish
Source: Zoological Lett. 2025 Jan 3;11:1. doi: 10.1186/s40851-024-00247-8 (PMC11697752; doi:10.1186/s40851-024-00247-8)
Supplement: Supplementary file 4 — Supplementary Material 4 [file 40851_2024_247_MOESM4_ESM.pdf]

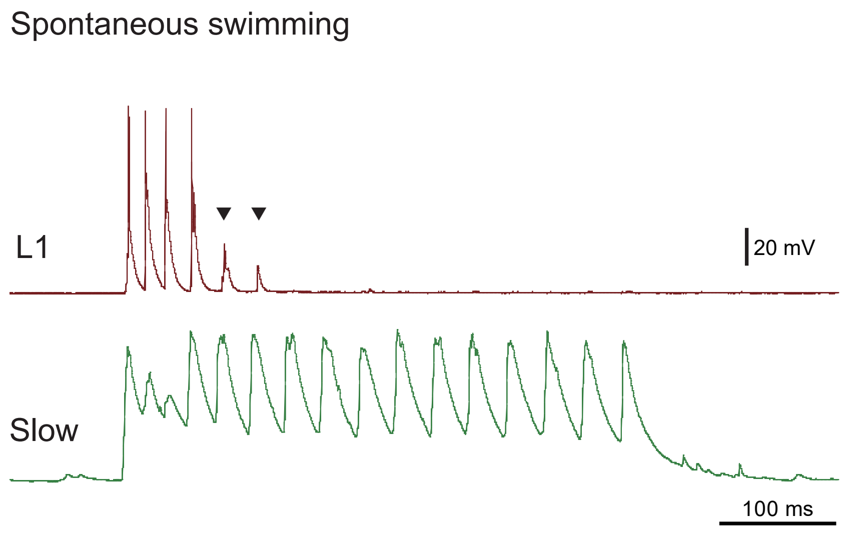

Additional File 4

Paired electrophysiological recordings between a slow muscle and an L1 fast muscle

Each peak in the slow muscle trace represents muscular activity during a spontaneous fictive swim bout. In the initial phase of the bout, the L1 fast muscle displayed spiking activity, which transitioned into subthreshold endplate potentials (EPPs) as indicated by the arrowhead. In the later phase of the bout, the L1 fast muscle ceased to exhibit any EPPs.
