# Supplementary material for: Spatially ordered recruitment of fast muscles in accordance with movement strengths in larval zebrafish
Source: Zoological Lett. 2025 Jan 3;11:1. doi: 10.1186/s40851-024-00247-8 (PMC11697752; doi:10.1186/s40851-024-00247-8)
Supplement: Supplementary file 5 — Supplementary Material 5 [file 40851_2024_247_MOESM5_ESM.pdf]

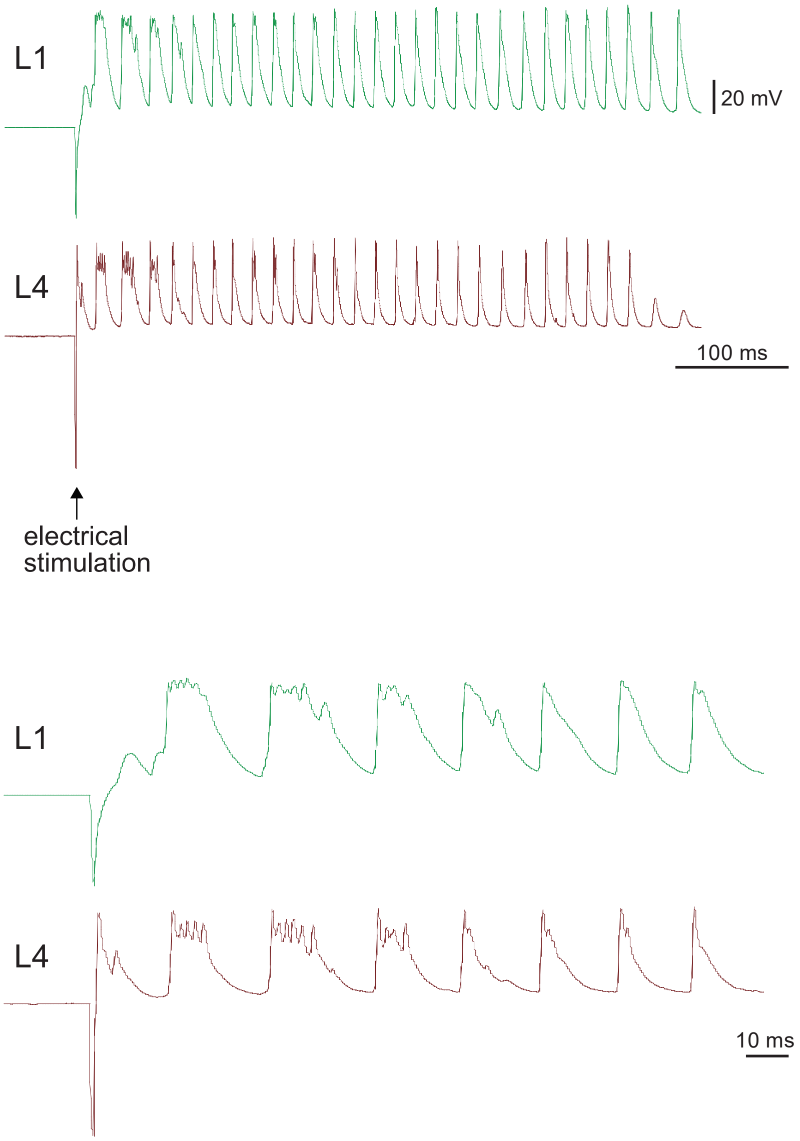

Additional File 5
Paired electrophysiological recordings between L1 and L4 fast muscles
The bottom panel provides a close-up view. In several swim cycles immediately following electrical stimulation, both L1 and L4 fast muscles exhibit multiple spikes per cycle.
